# Dental unit water content and antibiotic resistance of *Pseudomonas aeruginosa* and *Pseudomonas* species: a case study

**DOI:** 10.1080/20002297.2022.2107316

**Published:** 2022-08-23

**Authors:** M. Tesauro, M. Consonni, I. Grappasonni, G. Lodi, R. Mattina

**Affiliations:** a Department of Biomedical, Surgical and Dental Sciences, Laboratory of Environmental Hygiene, Coordinating Research Centres Episomi University of Milan, University of Milan, Milan, Italy; b School of Medicinal and Health Products Sciences, University of Camerino, Camerino, Italy

**Keywords:** Dental unit water line, *Pseudomonas aeruginosa*, *Pseudomonas* spp., antibiotic resistance, water microbiology, water safety plan

## Abstract

**Background:**

Many studies consider the contamination of dental unit waterlines (DUWLs), but few of them have studied the possible presence of antibiotic resistant *Pseudomonas aeruginosa* in the DUWLs.

**Aims:**

Investigation of the presence of *P. aeruginosa* and *Pseudomonas* spp. strains in DUWLs and evaluation of their resistance to six antibiotics (ceftazidime, netilmicin, piperacillin/tazobactam, meropenem, levofloxacin, colistin sulfate) at a public dental clinic in Milan, Italy.

**Results:**

Dental units were contaminated by *P. aeruginosa* with loads of 2–1,000 CFU/L and were mainly located on the mezzanine floor, with a range of 46–54%, while *Pseudomonas* spp. were primarily found on the first and second floors, ranging from 50 to 91%. *P. aeruginosa* was antibiotic resistant in 30% of the strains tested, and*Pseudomonas* spp. in 31.8% . Cold water from controls was also contaminated by these microorganisms.

**Conclusion:**

Monitoring antibiotic resistance in the water and adopting disinfection procedures on DUs are suggested within the Water Safety Plan.

## Introduction

The dental unit (DU) conveys water to the handpieces through a complex network of tubes to cool the teeth and instruments during dental treatments with high-speed rotating instruments and ultrasonic scalers, to irrigate the operating field or provide water for mouth rinsing from the fountain.

This complex network, the so-called Dental Unit Waterlines (DUWLs) (DUWLs), consists of valves, connectors and about 6 m of plastic pipes (usually polyurethane or polyvinyl) with a diameter of 0.5–2 mm. In the tubes, the water flows at different speeds, faster in the center, slower towards the walls until it reaches zero in contact with them.

This hydrodynamic phenomenon, combined with water stagnation due to standstill of the dental units at night, on weekends, during vacation periods and a water temperature around 20°C, favors the formation of biofilm.

This layer, formed by microorganisms (bacteria, fungi, protozoa,protozoa and viruses) trapped in a matrix of extracellular substances, mostly polysaccharides and proteins, allows the microbes to survive, to resist disinfection treatment and, if conditions are favorable, to multiply [1].

Therefore, DUWLs may be contaminated by water-borne microorganisms (environmental microorganisms), both free and sessile in the biofilm, originating from the municipal water supply piped into the dental unit. Microbial contamination can also derive from germs in patients’ oral cavities through the suck-back of patients’ saliva resulting from the aspirating effect of the rotating instruments at the end of their use, which causes a back-contamination of the water [2,3].

Many micro-organisms have been found in water samples from DUWLs such as *Streptococcus* spp., *Staphylococcus* spp., *Enterococcus* spp., *Enterobacteriaceae, Pseudomonas aeruginosa, Legionella pneumophila, Aeromonas* spp., *Acinetobacter* spp. and other environmental microorganisms, but also viruses and fungi [4,5].

Therefore, the DU can become an ecological niche for microorganisms naturally present in the environment and oral cavity. In dentistry, the infective risk is mainly focused on blood-borne infections and only secondly on those originating from environmental microorganisms, the so-called airborne and waterborne infections, due to the exposure to contaminated aerosols from high-speed rotating instruments that use cooling water or from water ingestion/contact.

Some of these microorganisms are characterized by low pathogenicity, others can be opportunistic, such as *P. aeruginosa*, which is considered one of the major causes of hospital-acquired infections. It belongs to the ‘ESKAPE’ pathogens (*Enterococcus faecium, Staphylococcus aureus, Klebsiella pneumoniae, Acinetobacter baumannii, P. aeruginosa* and *Enterobacter* species), which are extremely important for their impact on hospital infections and their ability to ‘escape’ the activity of antimicrobial drugs.

Antimicrobial resistance occurs when bacteria, viruses, fungi and parasites change over time and no longer respond to medicines making infections harder to treat and increasing the risk of disease spread, severe illness and death. As a result of drug resistance, antibiotics and other antimicrobial medicines become ineffective and infections become increasingly difficult or impossible to treat [6].

*P. aeruginosa* is frequently resistant to several antibiotics and considered ‘critical’ in the WHO’s priority pathogens list for research and development of new antibiotics. *P. aeruginosa* is characterized by a remarkable intrinsic resistance to several antibiotics, in addition to a resistance acquired through chromosomal mutations and acquisition of androgen receptor (AR) genes [7].

In the literature, there are many studies on the contamination of dental units by *P. aeruginosa* [8–11], but only a few of them studied the possible presence of antibiotic resistant *P. aeruginosa* in the DUWLs [12–14]. The aim of this work is to monitor the presence of *P. aeruginosa* and *Pseudomonas* spp. strains in the DUWLs and evaluate their resistance to 6 six antibiotics (ceftazidime, netilmicin, piperacillin/tazobactam, meropenem, levofloxacin, levofloxacin and colistin sulfate) in a public dental clinic in Milan. The increase in resistance against these antibiotics in the clinical practice is well known [15,16], therefore we wanted to learn more about the diffusion of the problem and the antibiotic resistant properties in a microorganism frequently present in the water and particularly in the DUWLs.

## Methods

The study was conducted in the Dental Clinic of a large hospital in Milan. The building was constructed in the late ‘90s and hosts 53 DUs, distributed over three floors, organized in open space ambulatories with several DUs and single ambulatories with only a dental unit. On the mezzanine floor floor, there are 13 DUs, seven of which are reserved for pediatric dental care and six are dedicated to adult dental care, in agreement with the National Health Service (NHS). On the first and the second floors floors, there are 20 DUs each, the first floor being dedicated to adult dental care in agreement with NHS, and the second floor for private practice. An operating room is present on the second floor.

Monitoring included sampling water from 44 DUs in service and four controls (cold water from common service taps, one control every 11–13 DUs), and precisely 13 DUs and one control on the mezzanine floor, 20 DUs and two controls on the first floor and 11 DUs, including the operating room, and one control on the second floor. The remaining DUs have been excluded because they were out of use during the survey period.

All DUs date back to the late 1990s, except for those reserved for pediatrics, which were renovated during the previous two years. None of the DUWLs are treated with chlorine products or other biocides, while surfaces and the handpieces are routinely treated with disinfectants between patients or are autoclaved.

The survey was performed between January and February 2020, before the pandemic period, on two different days of the week, on Monday morning, before beginning weekly activities, and on late afternoon Thursday or late morning Friday, at the close of weekly activities.

Using sterile bottles with thiosulfate (Italian LP), sampling was performed by collecting 200 mL from each handpiece of the dental unit before use (micromotors, scalers, scalers, and water-air syringe) and from the drinking fountain, for a total of 1 L of water. At the same time, an additional liter of cold water from the control taps was collected.

The samples were transported refrigerated to the Lab of Environmental Hygiene of the University of Milan, Italy and immediately analyzed.

Samples were processed according to UNI EN 12780:2002. Briefly, three aliquots of each sample (100 ml, 250 ml, 650 ml) were filtered using cellulose acetate membranes with 0.45 µm pore diameter (Millipore). The membranes were placed on Pseudomonas Cetrimide Agar (Oxoid) and incubated at 30°C for 24/48 h.

A total of 286 suspect colonies were isolated and tested firstly for fluorescence and oxidase activity. Fourteen strains were eliminated because of contaminations by molds and the remaining 272 strains were then sent for DNA extraction (GenElute Bacterial Genomic DNA Kits, Sigma Aldrich).

A qualitative Polymerase Chain Reaction (PCR) for the identification of the species *P. aeruginosa* was performed on the 272 extracted DNA samples. A 504 bp fragment (L lipoprotein, *OprL* gene) was amplified using primers PAL1, ATGGAAATGCTGAAATTCGGC and PAL2, CTTCTTCAGCTCGACGCGACG (Sigma-Aldrich).

The following cycles were performed: one cycle at 94°C for 5 min, 30 cycles at 94°C for 1 min, one cycle at 56°C for30for 30 s, one cycle at 72°C for30for 30 s, final extension cycle at 72°C for 7 min.

Negative samples for *P. aeruginosa* were then tested to identify species of the genus *Pseudomonas*. A fragment of 990 bp (*16S rRNA* gene) was amplified using the primers Ps-for GGTCTGAGGATGATCAGT and Ps-rev TTAGCTCCACCTCGCGGC (Sigma-Aldrich).

Electrophoretic runs were performed, in both cases, on 2% agarose gels.

To test antibiotic resistance, the E-test (BioMerieux), a ready-to-use antibiogram test with strips for MIC determination on a concentration gradient, was chosen. This is a quantitative method that allows determining the minimum concentration of an antibiotic that is capable of inhibiting bacterial growth. For this purpose, paper strips impregnated with antibiotic at known and decreasing concentration are used, which reflect the dilutions used in conventional methods for determining MIC. One side of the strip has a graduated reading scale, expressed in μg/ml, and an abbreviation specifying the type of the antimicrobial present.

Six bactericidal antibiotics widely used in clinical practice were chosen, piperacillin (concentration gradient 0.016–256 µml- – penicillins), levofloxacin (concentration gradient 0.002–32 µml – fluoroquinolones), netilmicin (concentration gradient 0.016–256 µml – aminoglycosides), CeftazimideCeftazidime (concentration gradient 0.016–256 µml – cephalosporins), colistin (concentration gradient 0.016–256 µml – polymyxins) and meropenem (concentration gradient 0.002–32 µml – carbapenems).

For each of the 272 strains, an 0.5 McFarland bacterial suspension in saline solution was prepared, 100 µl of this was spatulated onto 90 mm Mueller Hinton petri dishes (Oxoid) and allowed to dry under a biological hood. On each plate, two strips of antibiotic were placed head to tail and incubated at 35°C (±2°) for 16–20 h.

At the end of the incubation, MIC values were read at the point where the edge of the inhibition ellipse intersects the strip (rounding to the highest value). For the MIC interpretation, the EUCAST values (The European Committee on Antimicrobial Susceptibility TestingTesting) were used. Breakpoint tables for interpretation of MICs and zone diameters, version 8.1, 2018. http://www.eucast.org) were adopted. Data analyses regardedregarding occurrence of the dental unit contamination with *P. aeruginosa* and *Pseudomonas* spp., expressed as number of contaminated DUs or percentage, using the package Microsoft Excel 2016. Furthermore, occurrence of the antibiotic resistance as interpretation of the MIC was expressed as qualitative data, taking into account both single and multiple resistance patterns.

## Results

### Dental units contamination

*P. aeruginosa* was found in 10/44 DUs (22.7%) and 2/4 (50%) of controls, while *Pseudomonas* spp. were found in 23/44 (52.3%) and 3/4 (75%), both with a range of 2–1,000 CFU/L.

**Figure 1.**
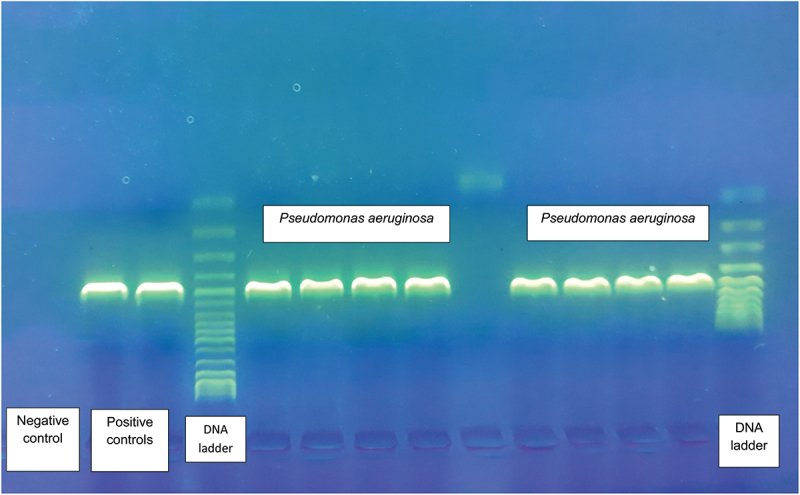
Agarose gel of PCR products of *P. aeruginosa* strains compared to positive and negative controls.

Colonies were selected on the basis of their morphology and colorcolor, and 286 of them were isolated from Cetrimide agar; subsequently, 14 strains were eliminated because of contamination with molds. In assessing the main biochemical properties of the remaining 272 strains, 163 strains were positive and 109 negative for the fluorescence tests, and 238 positive and 34 negative for oxidase, respectively. Therefore, DNA was extracted from 272 strains and qualitative PCR for *P. aeruginosa* was applied showing 70/272 (25.7%) positive samples; after the elimination of dubious samples, 198 were tested by PCR for identifying *Pseudomonas* spp., showing 105/198 (53.3%) positive strains (Figure 1).

**Figure 2.**
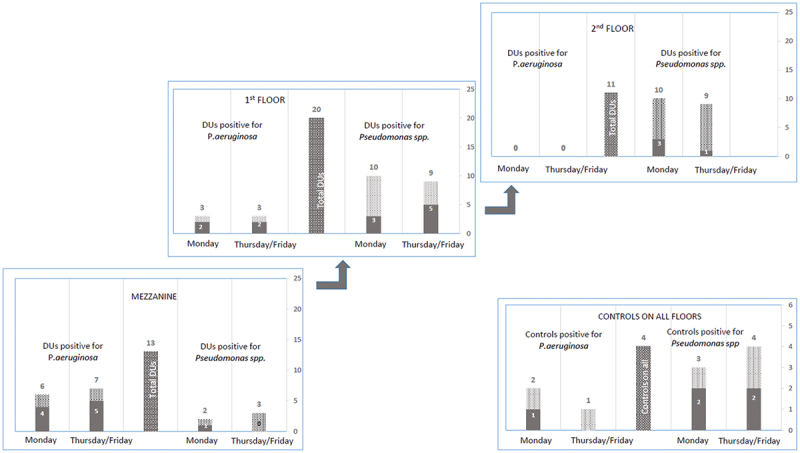
Number of DUs positive for *P. aeruginosa* and *Pseudomonas* spp. for both samples at each floor in light gray. In dark gray, number of DUs contaminated by antibiotic resistant strains. In the center column the total number of DU’s analyzed on each floor. The same synthesis for the control.

As shown in Figure 2, 6/13 DUs on Monday and 7/13 DUs on Thursday (range 46–54%) were contaminated by *P. aeruginosa* and mainly located in the pediatric unit on the mezzanine floor. The percentage decreased to 15 and 0% on the first and second floors with 3/20 and 0/11 DUs positive for *P. aeruginosa*. On the other hand, *Pseudomonas* spp. were primarily found in 10/20 DUs on the first floor and 10/11 DUs on the second floors, ranging from 50 to 91% of the analyzed DUs. Cold water from controls was contaminated by *P. aeruginosa* on the first floor and by *Pseudomonas* spp. on every floor.

### Antibiotic resistance – E-test

Subsequently, all 70/70 strains of *P. aeruginosa* and selected strains of *Pseudomonas* spp. (66/105) were tested for antibiotic resistance to piperacillin (PP), levofloxacin (LE), netilmicin (NC), Ceftazimide ceftazidime (TZ), colistin (CO) and meropenem (MP). We detected 94/136 strains (69.1%) sensible to all tested antibiotics, respectively respectively, 49/70 (70%) for *P. aeruginosa* and 45/66 (68%) for *Pseudomonas* spp.

In *P. aeruginosa* a total of 21/70 (30%) strains were resistant, mainly to colistin (15/70 strains, 21.4), while *Pseudomonas* spp. were resistant in 21/66 strains (31.8%), primarily against colistin (15/66 strains, 22.7%). Considering both *P. aeruginosa* and *Pseudomonas* spp., the most frequent resistance was confirmed against colistin (30/136 strains, 22%), followed by piperacillin (11/136 strains, 8%) and ceftazidime (8/136 strains, 5.9%) (Table 1).

**Table 1.** Summary of the resistance phenotype of the analyzed strains.

Resistance phenotype	*P. aeruginosa* N. isolates	*Pseudomonas* spp. N. isolates
CO	9	12
CO, MP		1
LE, NC, CO	1	
MP	1	1
PP	4	
PP, CO	2	
PP, NC		2
PP, NC, CO		1
PP, TZ		1
PP, TZ, CO	1	
TZ	1	2
TZ, CO	2	1
Total number of isolates	70	66

PP = piperacillin, LE = levofloxacin, NC = netilmicin, TZ = Ceftazimide ceftazidime, CO = colistin, MP = meropenem.

Regarding the multi-resistance properties, we evidenced that the percentage decreased from 5.7 and 7.5 for two antibiotics to 2.9 and 1.5 for three antibiotics respectively antibiotics, respectively, in *P. aeruginosa* and *Pseudomonas* spp. We did not observe resistance to four, five and six antibiotics contemporarily.

Interestingly, multi-resistance phenotype involved levofloxacin-netilmicin-colistin in one isolate, piperacillin-colistin in two of them and piperacillin-ceftazidime-colistin in the last case for *P. aeruginosa* strains. In *Pseudomonas* spp., the multi-resistance regarded colistin-meropenem, Pipecillin-netilmicin-colistin, piperacillin-Ceftazimide, Ceftazimide -colistin in one case, respectively, and Pipecillin-netilmicin in two isolates.

Antibiotic resistant *P. aeruginosa* strains were particularly observable on the mezzanine, in 4/6 DUs and 5/7 DUs, and the first floor with 2/3 DUs (67–71%). Otherwise, *Pseudomonas* spp. showed this property with less frequency (11–56%), but mostly at the first floor with 3/10 and 5/9 DUs. Cold water from controls was contaminated by antibiotic resistant *P. aeruginosa* at the first floor and by resistant *Pseudomonas* on every floor (Figure 2 in dark gray).

Editor’s note: In all 4 figures, it has been written *Pseudomonas* spp. or spp spp. is correct but should not be written in Italics.

## Discussion

The presence of long narrow-bore tubing, inconsistent flow rates, and oral fluid retraction can promote bacterial growth and biofilm development in the dental unit waterlines. Among other health risks due to the dental practice, dental health care personnel and patients could be exposed to risk derived by the contact, ingestion or inhalation of sessile or planktonic waterborne microorganisms, such as *Legionella* and *P. aeruginosa*. Therefore, DU water quality has to be guaranteed, setting a limit of 200 CFU/mL, the same threshold established for dialysate fluid [17].

According to the previous guidelines on the quality of water intended for human consumption (98/83/EC), drinking water should not be positive for *P. aeruginosa*. The recent drinking water directive 2020/2184 introduces the research on *Legionella* and surprisingly excludes *P. aeruginosa*, creating a regulatory vacuum with regard to this microorganism, which is an important opportunistic pathogen and remains a threat both in clinical practices, especially for immunosuppressed older people, smokers, pregnant women, and in ecological niches.

*P. aeruginosa* is one of the ‘ESKAPE’ pathogens and intrinsically resistant to most antimicrobial agents due to its low outer membrane permeability or its capability to extrude them through an efflux pump. Otherwise, *P. aeruginosa* has an adaptive resistance induced by the presence of antibiotics or other environmental factors such as biocides, pH, anaerobiosis, as well as social bacterial activities like swarming motility or biofilm formation [7,18]. It is commonly believed and demonstrated that inappropriate use of antibiotics has promoted the diffusion of antibiotic resistance in most microorganisms all over the world, included *Pseudomonas*, and actions are needed to prevent a post-antibiotic era in which common infections could again kill.

The knowledge of the spread of the phenomenon and against which antibiotics there is more resistance are among the first steps to be taken in the prevention activity and surveillance, along with their appropriate use. Our objective was to verify how much *P. aeruginosa* or *Pseudomonas* spp. are diffused in DUWLs and the antibiotic resistance properties they acquire in a clinical context, like a dental ward, but on an environmental matrix, such as the water of the DUs.

As we showed in the results, *P. aeruginosa* was found in quite 20% of the DUs analyzed and *Pseudomonas* spp. in percentages even greater, with a marked difference on different floors, characterized by a decrease of the number of contaminated DUs from the mezzanine to the second floor for *P. aeruginosa* and, on the contrary, an increase for the other microorganisms. It is difficult to understand the causes of these differences between floors, because all DUs were of the same period of manufacture, with the exception of the mezzanine with more recent DUs in the pediatric ward, and no water treatment is carried out on any of them.

We can only note two differences: 1) the type of patients treated on the different floors, as the DUs on the mezzanine floor were dedicated to children, while those on the upper floors were exclusively for adults, and 2) the difference in the age of manufacture of the dental units themselves, that, which could justify a different degree of colonization by microorganisms of human and environmental origin in the biofilm.

In 2014, a similar work published by Güngör et al. [19] reported that *P. aeruginosa* and *Pseudomonas* spp. were detected in 6% and 26% of the dental units analyzed in their study, showing a better condition. In another study, Al-Hiyasat et al. [20], described that in a dental education center *P. aeruginosa* was positive in 86.7% and 73.3% of the DUWLs at the beginning of the week and after flushing for two min minutes, respectively. Other authors [21 and 22] stated that 91% and 16% of the Gram-negative bacteria isolated from DUs were *Pseudomonas* spp. In a microbiological environmental investigation carried out in ten dental clinics in Italy [23]. *P. aeruginosa* was found in 2.38% (7/294) of tap water samples and in 20.06% (59/294) of DUWLs samples, with results comparable to those of our study.

Cristina et al. [12], observed that, out of the 4,500 water samples taken in various healthcare facilities, 30.11% of *P. aeruginosa* isolates came from DUs and 0.40% from inlet water samples. We also observed contamination by these microorganisms in the input water. This phenomenon is a strong indicator that the bacteria might have contaminated the DUs through the input water, thereafter; thereafter, the microorganism found an ideal ecological niche that allowed its adaptation, colonization, and multiplication.

Regarding the resistance against one or more antibiotics, we found several microorganisms resistant to different antibiotics, mainly to colistin and piperacillin, and the multidrug resistance was confirmed by other studies [12, 13].

The World Health Organization [6] considers *P. aeruginosa* as a critical microorganism, especially for the resistance to third generation cephalosporins and carbapenems. In our study the observed resistance against ceftazidime and meropenem was about 6% for the first and 1.4–3% for the second, revealing that the situation was not alarming but should be kept under observation. ECDC annual reports revealed that *P. aeruginosa* was the most frequently isolated microorganism in ICU-acquired infections and evidenced antimicrobial groups to be taken under surveillance [24 and 15], as the Italian Ministry of Health does periodically [16].

Considering antibiotic resistance as one of the most important public health issues in both human and veterinary medicine in the present and future, the strategy ‘One Health’ suggested by WHO, could help constrain the spread of the phenomenon, designing and implementing programs, policies, legislation and research in which multiple sectors communicate and work together to achieve better public health outcomes.

Regarding the diffusion of antibiotic resistant *P. aeruginosa* in water circulating in DUs, actions to prevent it should be suggested. The application of the Water Safety Plan [25] is the most adequate suggestion, encompassing the identification and assessment of hazards, hazardous events, risks and existing control measures. The constant microbial monitoring of *P. aeruginosa* and its antibiotic resistance pattern in the water is an action to implement the knowledge of the spread of this microorganism in the water network and to better understand where reservoirs of resistant *P. aeruginosa* are [26]. This will allow for adopting disinfection procedures on DUs, water treatments and maintenance of the entire water network through periodic monitoring of the water system.

To date, there is no definitive disinfection practice that has solved the problem of DUWL contamination. Several studies evidenced the encouraging results deriving from treatments with different disinfectants/products. Baudet et al. [27], reported the experience with the Biofilm-Removing-System® (BRS®) and Alpron®/Bilpron® disinfectant solutions for six years in a French University Hospital, where 99.8% of the samples were compliant with extended microbiological level, and there was no detection of pathogenic bacteria like *Legionella* sp. and *P. aeruginosa*. Furthermore, good evidence of antimicrobial efficacy of silver with hydrogen peroxide on diverse microorganism present in DUWLs was observed but there was insufficient evidence on the application of silver nanoparticles as an efficient material to control the biofilms in DUWLs [28]. Low-concentrated ozonized water is bactericidal against heterotrophic bacteria biofilms and it is not harmful to DUs, and plasma sterilization, which is part of electrochemically activated water, effectively reduces bacterial contamination and reduces biofilms in dental unit waterlines, but both studies are at an experimental phase [29,30]. Other authors tried to identify an improvement program in four steps instead of a single intervention in a medical center, reaching interesting results: discharge of DUWLs for 5 min in the morning before clinical service to flush out the water left in the pipeline overnight; weekly disinfection of the handpiece connector with 75% alcohol; monthly disinfection of the water supply system and pipeline; and establishment of DU maintenance work standards and staff education and training [31].

In addition to these interventions, an active *P. aeruginosa* infection surveillance is also necessary. This is a strong limitation on our research in which environmental data could not have a relationship with the clinical data and, therefore, we can only speculate about the health risks for patients and operators. Another limitation is that data presented in this work refer to a single monitoring campaign and a new series of analysis is needed. All these possible actions unfortunately lack in our monitoring site, and we want to support the operators in the implementation of them.

## Protection of research participants

No humans and no animals were involved in the research.

## Health and safety

All mandatory laboratory health and safety procedures have been complied within the course of conducting the experimental work reported in the present paper.
